# *Marseilleviridae* Lineage B Diversity and Bunch Formation Inhibited by Galactose

**DOI:** 10.1264/jsme2.ME20139

**Published:** 2021-02-20

**Authors:** Keita Aoki, Sho Fukaya, Haruna Takahashi, Mio Kobayashi, Kenta Sasaki, Masaharu Takemura

**Affiliations:** 1 Laboratory of Biology, Graduate School of Mathematics and Science Education, Tokyo University of Science, Kagurazaka 1–3, Shinjuku, Tokyo 162–8601, Japan; 2 Laboratory of Biology, Faculty of Science Division I, Tokyo University of Science, Kagurazaka 1–3, Shinjuku, Tokyo 162–8601, Japan

**Keywords:** *Marseilleviridae*, hokutovirus, tokyovirus, bunch, diversity

## Abstract

*Marseilleviridae* is a family of large double-stranded DNA viruses that is currently divided into five subgroups, lineages A–E. Hokutovirus and kashiwazakivirus, both of which belong to lineage B, have been reported to induce host acanthamoeba cells to form aggregations called “bunches”. This putatively results in increased opportunities to infect acanthamoeba cells, in contrast to lineage A, which has been reported to not form “bunches”. In the present study, we isolated 14 virus strains of the family *Marseilleviridae* from several Japanese water samples, 11 of which were identified as lineage B viruses. All 11 lineage B strains caused infected amoeba cells to form bunches. We then investigated the involvement of monosaccharides in bunch formation by amoeba cells infected with hokutovirus. Galactose inhibited bunch formation, thereby allowing amoeba cells to delay the process, whereas mannose and glucose did not. A kinetic image analysis of hokutovirus-infected amoeba cells confirmed the inhibition of bunch formation by galactose. The number of hokutovirus-infected amoeba cells increased more rapidly than that of tokyovirus-infected cells, which belongs to lineage A. This result suggests that bunch formation by infected amoeba cells is advantageous for lineage B viruses.

The family *Marseilleviridae* is a group of large double-stranded DNA viruses belonging to nucleocytoplasmic large DNA viruses (NCLDVs) that comprise icosahedral viruses with particle sizes of approximately 250 nm and genome sizes of 350–380‍ ‍kb (Boyer *et al.*, 2009; Colson *et al.*, 2013; Takemura, 2016; Aoki *et al.*, 2019). The founder virus of the family *Marseilleviridae* is *Marseillevirus marseillevirus*, which was originally isolated from the water of a cooling tower in Paris, France (Boyer *et al.*, 2009) and is a member of the so-called “giant viruses”. Since the discovery of the founder strain, many family members of *Marseilleviridae* have been isolated, not only from aquatic environments, but also from organisms ranging from humans to insects (Colson *et al.*, 2013). For example, melbournevirus (Doutre *et al.*, 2014), Cannes 8 virus (Aherfi *et al.*, 2013), senegalvirus (Legier *et al.*, 2012), tokyovirus (Takemura, 2016), *Marseilleviridae shanghai* (unpublished), lausannevirus (Thomas *et al.*, 2011), Port-miou virus (Doutre *et al.*, 2015), noumeavirus (Fabre *et al.*, 2017), kurlavirus (Chatterjee and Kondabagil, 2017), tunisvirus (Aherfi *et al.*, 2014), insectomime virus (Boughalmi *et al.*, 2013), Brazilian marseillevirus (Dornas *et al.*, 2016), golden marseillevirus (Dos Santos *et al.*, 2016), and kashiwazakivirus, hokutovirus, and kyotovirus (Aoki *et al.*, 2019) have all been isolated to date. The family *Marseilleviridae* is currently classified into five lineages, from A to E (Chatterjee and Kondabagil, 2017; Fabre *et al.*, 2017; Aoki *et al.*, 2019).

We recently isolated 15 marseillevirus strains from aquatic Japanese environments and named them kashiwazakivirus 1 through 6, hokutovirus 1 and 2, and kyotovirus 1 through 7 (Aoki *et al.*, 2019). Twelve out of the 15 marseillevirus strains have major capsid protein (MCP) genes with sequences that slightly differ from each other. According to a molecular phylogenetic analysis of MCP and D5-like helicase-primase genes, kashiwazakiviruses and hokutoviruses both belong to lineage B of *Marseilleviridae*, while kyotoviruses belong to lineage A (Aoki *et al.*, 2019). All kashiwazakivirus and hokutovirus strains at a multiplicity of infection (MOI) of approximately 4 were found to induce amoeba cells to form aggregates called “bunches” during the early stage of viral infection, similar to that observed for tupanvirus-infected amoebae (Abrahão *et al.*, 2018; Oliveira *et al.*, 2019). Although bunch formation was delayed in amoeba cells infected with fewer of these viruses (MOI=0.1), bunch sizes were larger than those observed at MOI=4 (Aoki *et al.*, 2019). On the other hand, kyotovirus-infected amoeba cells failed to exhibit bunch formation, which indicated that the process is a phenomenon specific to the *Marseilleviridae* lineage B viruses kashiwazakivirus and hokutovirus. The precise mechanisms underlying bunch formation currently remain unclear. To elucidate the biological significance of bunch formation, it is important to confirm whether it is a lineage B-specific feature and analyze new viruses belonging to lineage B accordingly.

In the present study, we isolated several additional viruses belonging to lineages A and B, as well as other putative members of *Marseilleviridae* from various environments in Japan. The results obtained revealed the diversity and specific features of lineage B, which induces bunch formation in amoeba. We also investigated the mechanisms underlying bunch formation in amoeba cells infected with hokutovirus and identified a galactose-mediated mechanism responsible for the induction of bunch formation.

## Materials and Methods

### Cell culture and viruses

*Acanthamoeba castellanii* (Douglas) Neff cells (ATCC 30010^TM^) were cultured in proteose peptone-yeast extract-glucose (PYG) medium as previously described (Takemura, 2016; Aoki *et al.*, 2019; Yoshikawa *et al.*, 2019). Stocks of previously isolated marseilleviruses, hokutovirus 1, and tokyovirus were used in the present study (Takemura, 2016; Aoki *et al.*, 2019).

### Virus isolation and titration

Water samples were collected from five sampling locations in Japan, namely, the Koaze River in Saitama Prefecture, the Oosagami Regulating Pond in Saitama Prefecture, the mouth of the Arakawa River in metropolitan Tokyo named Shinsuna, the mouth of the Sabaishi River in Niigata Prefecture, and a small reservoir near Hokuto town of Kashiwazaki city in Niigata Prefecture. In all samples, 4.5‍ ‍mL of water was combined with 4.5‍ ‍mL of 2× PYG medium, 360‍ ‍μL of an antibiotic solution containing ampicillin, penicillin-streptomycin, and amphotericin B, and 100‍ ‍μL of the amoeba cell suspension. The cytopathic effects (CPE) of amoeba cells were observed using a Nikon Eclipse TS100 phase-contrast microscope. After observing the CPE of cultured amoeba cells, putative viruses were propagated and cloned using a serial 10-fold dilution method, as previously described (Aoki *et al.*, 2019). Cloned viruses were collected by centrifugation at 8,000×*g* at 4°C for 35‍ ‍min and were then resuspended in sterile phosphate-buffered saline (PBS). The titration of virus preparations was performed using a tissue culture infectious dose 50% (TCID_50_) calculator and MOI was assessed, as previously described (Aoki *et al.*, 2019). Isolated viruses were evaluated as potential members of *Marseilleviridae* based on a polymerase chain reaction (PCR) analysis using primers specific for *Marseilleviridae* MCP genes (Aoki *et al.*, 2019).

### Molecular phylogenetic analysis of MCP genes

After cloning, the genomic DNA of the isolated viruses was extracted using a NucleoSpin Tissue XS Kit (Macherey-Nagel GmbH and Co. KG) according to the manufacturer’s protocol (Aoki *et al.*, 2019). Full-length MCP genes were amplified and sequenced using primers designed for the *Marseilleviridae* family as described previously (Aoki *et al.*, 2019). The nucleotide sequences of the MCP genes of isolated viruses and those of known members of the *Marseilleviridae* family obtained from the National Center for Biotechnology Information (NCBI) nucleotide sequence database were aligned and a maximum-likelihood phylogenetic tree was constructed using Molecular Evolutionary Genetics Analysis (MEGA) X software (Kumar *et al.*, 2018), as described previously (Aoki *et al.*, 2019). The full-length MCP gene sequences of newly isolated Marseilleviridae strains have been submitted to the DNA Data Bank of Japan (DDBJ; accession numbers: oosagami B5, LC572164; oosagami C9, LC572165; oosagami E6, LC572166; koaze, LC572163; shinsuna B6, LC572161; Shinsuna C7, LC572162; yoshiyabu A1, LC572167; yoshiyabu C2, LC572168; yoshiyabu C11, LC572169; yoshiyabu E7, LC572170; yoshiyabu F1, LC572171; yoshiyabu G4, LC572172; sabaishi A2, LC572159; and sabaishi H11, LC572160).

### Monosaccharide inhibition of bunch formation

Bunch formation by acanthamoeba cells was evaluated with or without the addition of several monosaccharides. Acanthamoeba cells were cultured in PYG medium without glucose (PY medium) containing 500‍ ‍mM of the monosaccharides galactose, mannose, or glucose. Amoeba cells were then infected with hokutoviruses (MOI=1) and fluctuations in their average particle sizes were analyzed. Briefly, 45-sec time-lapse images of virus-infected amoeba cells cultured with monosaccharides were captured using all-in-one fluorescent microscopy with a BZ-X800/X810 microscope (Keyence). Kinetic analyses of cells were performed as previously described using a phase-contrast-based kinetic analysis algorithm for amoebae (PKA3) developed in our laboratory (Fukaya *et al.*, 2020).

### Hokutovirus-induced bunch formation compared with tokyovirus

Acanthamoeba cell cultures were monitored for bunch formation as previously described (Aoki *et al.*, 2019). Amoeba cells in each well of the culture plate were observed using all-in-one fluorescent microscopy with a BZ-X800/X810 microscope (Keyence) as previously described at 12, 24, and 48 hpi (hours post infection) with hokutovirus 1 or tokyovirus (MOI=1) (Aoki *et al.*, 2019).

## Results

### Bunch formation was a *Marseilleviridae* lineage B-specific property

**Fig. 1.**
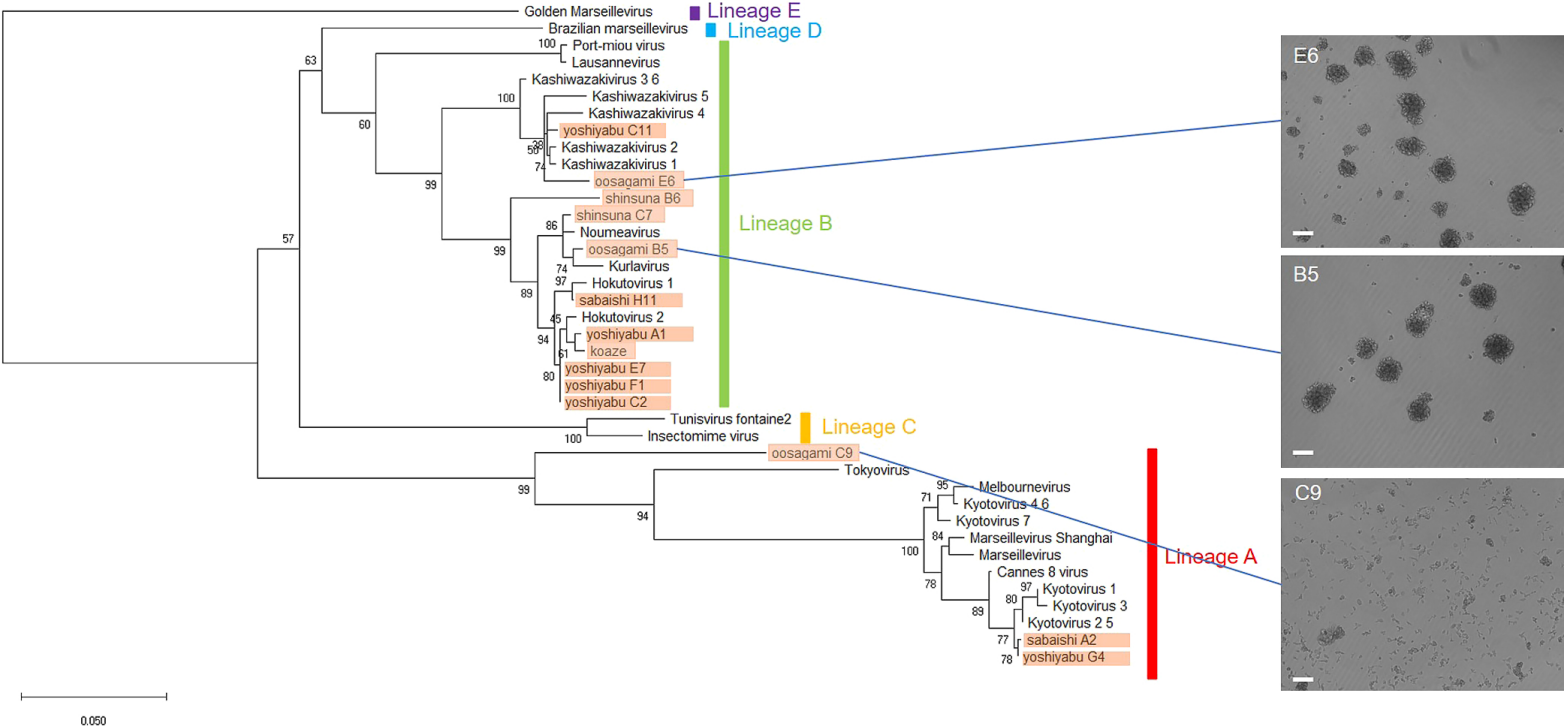
Molecular phylogenetic analysis of major capsid protein (MCP) genes of the family *Marseilleviridae*, including newly isolated strains in the present study. Strains in orange are new *Marseilleviridae* viruses isolated in the present study. Phase-contrast microscopy images on the right show amoeba cells infected with isolated *Marseilleviridae* strains oosagami E6, B5, and C9 with or without the formation of “bunches”. The scale bar indicates 100‍ ‍μm.

In the present study, we isolated novel viruses of the family *Marseilleviridae* from five Japanese aquatic sampling locations. Among these sites, the small reservoir near Hokuto town of Kashiwazaki city in Niigata Prefecture was the same sampling location from which hokutovirus was isolated (Aoki *et al.*, 2019). Fourteen strains of the family *Marseilleviridae* were isolated from these five sampling locations, which was confirmed by sequencing and molecular phylogenetic analyses of the MCP genes of the new strains. Based on molecular phylogenetic analyses, 11 strains belonged to lineage B, two to lineage A, and one to an unknown lineage that was close to tokyovirus (Fig. 1 and S1). According to infection experiments, the 11 lineage B strains induced bunch formation by amoeba as described previously (Aoki *et al.*, 2019), whereas the three other strains, which did not belong to lineage B, did not (Fig. 1). Although we have not yet tested lineage C, D, or E, these results suggest that bunch formation is a lineage B-specific property of *Marseilleviridae* and not a characteristic of the viruses of lineage A.

### Galactose inhibited bunch formation

**Fig. 2.**
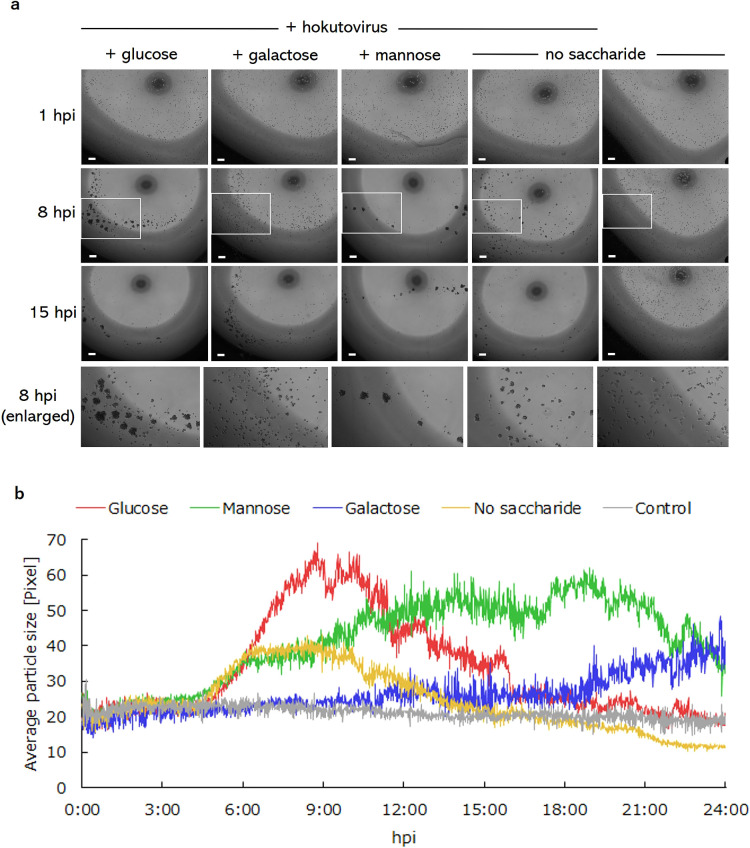
Effects of monosaccharides on “bunch” formation by hokutovirus-infected amoeba cells. (a) Hokutovirus-infected amoeba cells were cultured under the condition of 500‍ ‍mM galactose, mannose, or glucose. The positive control is hokutovirus-infected amoeba cells cultured without any added monosaccharides. The negative control is amoeba cells without hokutovirus or any added monosaccharides. h.p.i., hours post infection; Gal, galactose; Man, mannose; and Glc, glucose. Black spots and dark areas shown in each photograph were caused by the fluctuated light captured by phase-contrast processes using 96-well plates. Inbox areas in photographs of 8 hpi cultures were enlarged and shown below photographs of 15 hpi. The scale bar indicates 100‍ ‍μm. (b) Kinetic image analysis of hokutovirus-infected amoeba cells in the presence or absence of monosaccharides. Time-lapse images of hokutovirus-infected or healthy amoeba cells cultured with media supplemented with or without monosaccharides (glucose, mannose, or galactose) were captured using a BZ-X800/X810 all-in-one fluorescent microscope and kinetic image analyses of cells were performed using the PKA3 algorithm developed in our laboratory, as previously described (Fukaya *et al.*, 2020).

A previous study reported that mannose-binding protein (MBP) expressed on the amoeba cell membrane may be involved in bunch formation by amoeba cells infected with tupanvirus, a close relative of mimivirus, suggesting the involvement of monosaccharides in the bunch formation process (Oliveira *et al.*, 2019). Since PYG medium, which is commonly used to culture acanthamoeba, contains 0.1 M glucose, we used PY medium, which does not contain glucose, to avoid any effects of glucose on our inhibition experiments. As shown in Fig. 2a, the positive control (no saccharide) and hokutovirus-infected amoeba cells in the presence of mannose or glucose formed bunches at 8 hpi. In contrast, infected amoeba cells in the presence of galactose only formed small bunches at 15 hpi (Fig. 2a). These results clearly suggest that the addition of galactose to culture media inhibited bunch formation more than glucose, mannose, or the positive control, to which no saccharide was added (Fig. 2a). This inhibitory effect of galactose was reproduced in another experiment (Fig. S2).

According to our PKA3 algorithm-based kinetic image analysis of amoeba cells cultured in media containing sugars (Fukaya *et al.*, 2020), it was clear that the addition of galactose inhibited the increase in average “particle size” in both single and aggregated cells (Fig. 2b, Movie S1, and S2). The decrease in average particle sizes exhibited by cells cultured with glucose or the positive control was considered to be an artifact as the bunches were out of the field of view (Fig. 2b). This result indicated that the inhibition of bunch formation was caused by galactose and suggested that bunch formation by amoeba cells induced by infection with hokutovirus was a galactose-mediated interaction between amoeba cells. The results from the kinetic analysis were reproduced in another experiment (Fig. S3).

### Hokutovirus infection stimulated bunch formation around healthy cells

**Fig. 3.**
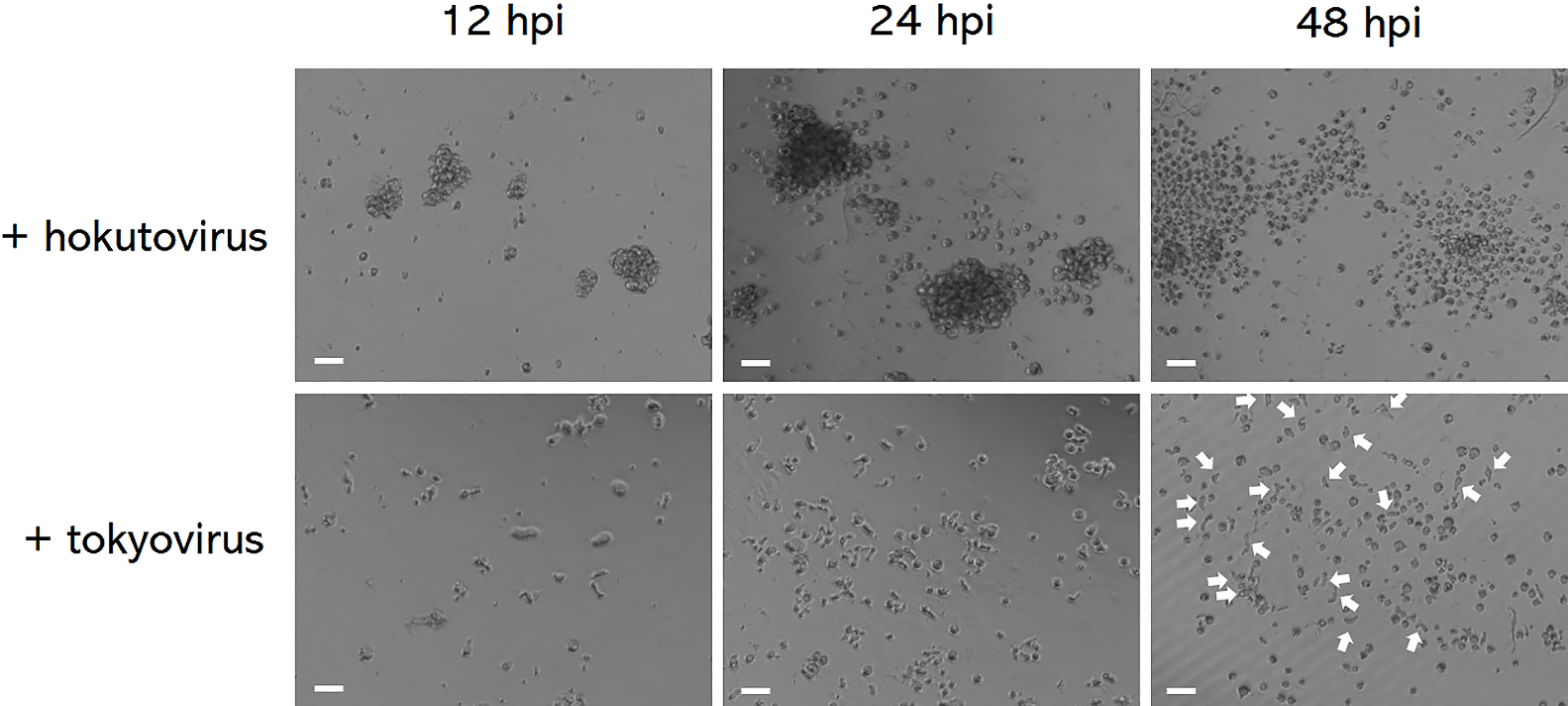
Effects of “bunch” formation on the ratios of amoeba cells showing cytopathic effects (CPE) and healthy adhered cells. Amoeba cells were infected with hokutovirus or tokyovirus at MOI=1 and cells were then observed 12, 24, and 48 hpi (hours post infection) using a BZ-X800/X810 all-in-one fluorescent microscope. The scale bar indicates 100‍ ‍μm.

We then counted and compared the ratio of cells exhibiting CPE (cell rounding caused by infection) among healthy adhered cells in microscopic fields of view. Final CPE caused by both hokutovirus and tokyovirus infections was primarily the rounding of amoeba cells. At 12 and 24 hpi, amoeba cells infected with hokutovirus at MOI=1 formed many bunches, which was in contrast to those infected with tokyovirus (Fig. 3). At 48 hpi, bunches formed by hokutovirus-infected amoebae had all collapsed and rounding cells had dispersed, resulting in almost all cells exhibiting CPE. In contrast, many adherent cells, which were not yet rounded, exhibited characteristics similar to those of tokyovirus-infected cells (Fig. 3, indicated by white arrows). The same pattern of cells with or without CPE was observed in wider fields of view (data not shown). These results suggested that bunch formation by infected amoeba cells was more advantageous for lineage B viruses than for lineage A viruses with respect to infection efficiency.

## Discussion

Viruses of the family *Marseilleviridae* are the smallest of the “giant viruses” among NCLDVs that infect *Acanthamoeba* spp. and have developed interesting strategies for propagation. Acanthamoeba engulf particles >500‍ ‍nm in diameter, such as mimiviruses, pandoraviruses, and pithoviruses; however, marseillevirus particles are only approximately 250‍ ‍nm and are not recognized by acanthamoeba cells as phagocytotic prey. Therefore, marseilleviruses have developed various strategies by which to be phagocytosed by acanthamoeba cells, such as the collective effects of particles leading to engulfment by amoeba cells. Marseilleviruses form giant vesicles containing many virus particles surrounded by membranes derived from the amoeba endoplasmic reticulum, which may stimulate phagocytosis in addition to the endocytotic invasion of single particles by amoeba cells (Arantes *et al.*, 2016). This strategy for being engulfed by amoeba cells may have provided evolutionary advantages to the members of *Marseilleviridae*, which consist of relatively smaller virus particles. Furthermore, at least one group of this family, lineage B, has developed unique behavioral characteristics that cause phenotypic changes in the host amoeba and the formation of bunches.

In the present study, we identified 14 new virus strains of the family *Marseilleviridae*, 11 of which belonged to lineage B and had the ability to induce bunch formation. These results clearly suggest that the induction of bunch formation by infected amoeba cells is a typical property of lineage B *Marseilleviridae* viruses. However, the mechanisms underlying bunch formation have not yet been elucidated. In the case of bunch formation by amoeba cells infected with tupanvirus, a close relative of mimivirus, MBP expressed on amoeba cells may be involved in the bunching of cells. Furthermore, tupanvirus itself may encode a MBP gene in its own genome (Abrahão *et al.*, 2018; Oliveira *et al.*, 2019). In a previous study, the addition of mannose to culture media inhibited bunch formation in tupanvirus-infected amoeba cells (Oliveira *et al.*, 2019). This finding suggests that bunch formation by tupanvirus-infected amoeba cells is mediated by cell-cell interactions involving a mannose recognition process. In the case of hokutovirus, it is also possible to inhibit or delay bunch formation by adding galactose to the culture media. Therefore, bunch formation by hokutovirus-infected amoeba cells may be mediated through cell-cell interactions involving a galactose recognition process, which is supported by the members of *Marseilleviridae* containing a galactose-binding protein (GBP) gene in their genomes. The alignment of the amino acid sequences of several marseillevirus GBP proteins revealed differences in the sequences of the respective N-terminal regions between lineage B and lineage A viruses (Fig. S4), which may have contributed to the different bunch induction abilities observed. Further studies are needed to elucidate the responsible sequences and underlying molecular mechanisms in the bunch formation process.

As shown in Fig. 3, bunch formation by infected amoeba cells may be advantageous for lineage B viruses because the ratio of amoeba cells exhibiting CPE at least two days after infection was higher than that of cells infected with lineage A tokyovirus. Bunches have been reported to contain not only rounding cells, but also healthy non-infected cells (Aoki *et al.*, 2019; Oliveira *et al.*, 2019). We noted the prominent movement of bunches induced by hokutovirus infection. Therefore, developing bunches appear to move using non-rounded cells and capture healthy amoeba cells. This property is also exhibited by tupanviruses (Oliveira *et al.*, 2019). Bunch formation may increase the opportunity for viruses to infect healthy amoeba cells because more amoeba cells became involved as bunching increased. Although providing more opportunities for infection is considered to increase the genetic diversity of viruses, it is difficult to conclude that the lineage B viruses of *Marseilleviridae* are more diversified than other *Marseilleviridae* lineages. Furthermore, bunch formation by amoeba cells demonstrated in the laboratory may rarely occur in nature because amoeba cells may not gather as closely under natural conditions, even though acanthamoeba is widely distributed worldwide. However, this type of strategy by lineage B marseilleviruses may induce host cells to form bunches and may play important roles in their evolution and diversification through as yet unknown mechanisms.

## Citation

Aoki, K., Fukaya, S., Takahashi, H., Kobayashi, M., Sasaki, K., and Takemura, M. (2021) *Marseilleviridae* Lineage B Diversity and Bunch Formation Inhibited by Galactose. *Microbes Environ ***36**: ME20139.

https://doi.org/10.1264/jsme2.ME20139

## Supplementary Material

Supplementary Material 1

Supplementary Material 2

Supplementary Material 3
