# Supplementary material for: Marseilleviridae Lineage B Diversity and Bunch Formation Inhibited by Galactose
Source: Microbes Environ. 2021 Feb 20;36(1):ME20139. doi: 10.1264/jsme2.ME20139 (PMC7966940; doi:10.1264/jsme2.ME20139)
Supplement: Supplementary file 1 — Supplementary Material 1 [file 36_20139_s1.pdf]

Supplementary Materials

Title:

Marseilleviridae lineage B diversity and bunch formation is inhibited by galactose

Authors:

Keita Aoki[1]*, Sho Fukaya[1]*, Haruna Takahashi[1], Mio Kobayashi[1], Kenta Sasaki[1], and Masaharu Takemura[1,2,]**

[1] Laboratory of Biology, Graduate School of Mathematics and Science Education, Tokyo University of Science, Kagurazaka 1-3, Shinjuku, Tokyo 162-8601, Japan

[2] Laboratory of Biology, Faculty of Science Division I, Tokyo University of Science, Kagurazaka 1-3, Shinjuku, Tokyo 162-8601, Japan

* These authors equally contributed to this work.

**Corresponding author: E-mail: giantvirus@rs.tus.ac.jp; Tel & Fax: +81-3-5228-8373

Supplementary materials include:

Supplementary Figure 1 (Figure S1)
Supplementary Figure 2 (Figure S2)
Supplementary Figure 3 (Figure S3)
Supplementary Figure 4 (Figure S4)
Supplementary Movie 1 (Movie S1)
Supplementary Movie 2 (Movie S2)

	1	2	3	4	5	6	7	8	9	10	11	12	13	14	15	16	17	18	19	20
1. oosagami_C9		99.88	99.87	99.86	99.87	99.86	99.86	99.86	99.86	99.87	99.87	99.86	99.86	99.83	99.84	99.85	99.84	99.86	99.85	99.86
2. Tokyovirus	99.88		99.88	99.89	99.88	99.87	99.87	99.87	99.87	99.89	99.89	99.88	99.88	99.85	99.86	99.84	99.84	99.85	99.84	99.84
3. Marseillevirus	99.87	99.88		99.97	99.99	99.98	99.97	99.98	99.98	99.98	99.98	99.98	99.98	99.84	99.84	99.83	99.83	99.84	99.84	99.84
4. Melbournevirus	99.86	99.89	99.97		99.98	99.96	99.97	99.97	99.97	99.99	99.98	99.97	99.97	99.83	99.83	99.82	99.82	99.85	99.84	99.85
5. Marseillevirus Shanghai	99.87	99.88	99.99	99.98		99.98	99.97	99.97	99.97	99.98	99.98	99.97	99.97	99.83	99.83	99.83	99.83	99.84	99.83	99.84
6. Cannes_8_virus	99.86	99.87	99.98	99.96	99.98		99.98	99.99	99.99	99.97	99.97	99.99	99.99	99.85	99.84	99.85	99.85	99.83	99.84	99.84
7. Kyotovirus_1	99.86	99.87	99.97	99.97	99.97	99.98		100	100	99.98	99.97	99.99	99.99	99.85	99.84	99.85	99.85	99.83	99.84	99.83
8. Kyotovirus_2_5	99.86	99.87	99.98	99.97	99.97	99.99	100		99.99	99.97	99.98	99.99	100	99.85	99.84	99.85	99.85	99.83	99.84	99.83
9. Kyotovirus_3	99.86	99.87	99.98	99.97	99.97	99.99	100	99.99		99.97	99.97	99.99	99.99	99.84	99.83	99.84	99.84	99.83	99.84	99.83
10. Kyotovirus_4_6	99.87	99.89	99.98	99.99	99.98	99.97	99.98	99.97	99.97		99.99	99.97	99.97	99.83	99.83	99.83	99.83	99.85	99.84	99.85
11. Kyotovirus_7	99.87	99.89	99.98	99.98	99.98	99.97	99.97	99.98	99.97	99.99		99.98	99.98	99.83	99.84	99.83	99.83	99.85	99.85	99.86
12. sabaishi_A2	99.86	99.88	99.98	99.97	99.97	99.99	99.99	99.99	99.99	99.97	99.98		100	99.85	99.84	99.85	99.85	99.84	99.84	99.84
13. yoshiyabu_G4	99.86	99.88	99.98	99.97	99.97	99.99	99.99	100	99.99	99.97	99.98	100		99.85	99.84	99.85	99.85	99.84	99.84	99.84
14. Tunisvirus_fontaine2	99.83	99.85	99.84	99.83	99.83	99.85	99.85	99.85	99.84	99.83	99.83	99.85	99.85		99.96	99.89	99.89	99.87	99.88	99.87
15. Insectomime virus	99.84	99.86	99.84	99.83	99.83	99.84	99.84	99.84	99.83	99.83	99.84	99.84	99.84	99.96		99.88	99.88	99.88	99.87	99.87
16. Port-miou_virus	99.85	99.84	99.83	99.82	99.83	99.85	99.85	99.85	99.84	99.83	99.83	99.85	99.85	99.89	99.88		100	99.91	99.92	99.91
17. Lausannevirus	99.84	99.84	99.83	99.82	99.83	99.85	99.85	99.85	99.84	99.83	99.83	99.85	99.85	99.89	99.88	100		99.91	99.92	99.91
18. sabaishi_H11	99.86	99.85	99.84	99.85	99.84	99.83	99.83	99.83	99.83	99.85	99.85	99.84	99.84	99.87	99.88	99.91	99.91		99.95	99.98
19. shinsuna_B6	99.85	99.84	99.84	99.84	99.83	99.84	99.84	99.84	99.84	99.84	99.85	99.84	99.84	99.88	99.87	99.92	99.92	99.95		99.96
20. shinsuna_C7	99.86	99.84	99.84	99.85	99.84	99.84	99.83	99.83	99.83	99.85	99.86	99.84	99.84	99.87	99.87	99.91	99.91	99.98	99.96	
21. koaze	99.87	99.85	99.84	99.85	99.84	99.83	99.83	99.83	99.82	99.84	99.85	99.83	99.83	99.87	99.87	99.91	99.91	99.99	99.96	99.98
22. oosagami_B5	99.86	99.84	99.84	99.85	99.84	99.83	99.83	99.83	99.83	99.85	99.85	99.84	99.84	99.87	99.87	99.91	99.91	99.98	99.96	99.99
23. oosagami_E6	99.87	99.86	99.86	99.85	99.85	99.85	99.85	99.85	99.85	99.85	99.85	99.85	99.85	99.89	99.89	99.92	99.92	99.95	99.93	99.93
24. yoshiyabu_A1	99.87	99.85	99.84	99.85	99.84	99.83	99.83	99.83	99.82	99.84	99.85	99.83	99.83	99.86	99.87	99.91	99.91	99.98	99.95	99.98
25. yoshiyabu C2	99.87	99.85	99.84	99.85	99.84	99.83	99.83	99.83	99.83	99.84	99.85	99.84	99.84	99.87	99.87	99.91	99.91	99.99	99.96	99.98
26. yoshiyabu_C11	99.87	99.86	99.85	99.84	99.84	99.85	99.85	99.85	99.84	99.84	99.85	99.85	99.85	99.88	99.89	99.92	99.92	99.95	99.92	99.93
27. yoshiyabu_E7	99.87	99.85	99.84	99.85	99.84	99.83	99.83	99.83	99.83	99.84	99.85	99.84	99.84	99.87	99.87	99.91	99.91	99.99	99.96	99.98
28. yoshiyabu_F1	99.87	99.85	99.84	99.85	99.84	99.83	99.83	99.83	99.83	99.84	99.85	99.84	99.84	99.87	99.87	99.91	99.91	99.99	99.96	99.98
29. Noumeavirus	99.86	99.84	99.84	99.85	99.84	99.83	99.83	99.83	99.83	99.85	99.86	99.84	99.84	99.87	99.87	99.91	99.91	99.98	99.96	99.99
30. Kurlavirus	99.86	99.84	99.84	99.84	99.83	99.83	99.83	99.83	99.83	99.84	99.85	99.84	99.84	99.88	99.88	99.92	99.92	99.97	99.96	99.98
31. Hokutovirus_1	99.87	99.85	99.84	99.85	99.84	99.84	99.83	99.83	99.83	99.85	99.85	99.84	99.84	99.87	99.88	99.91	99.91	99.99	99.95	99.98
32. Hokutovirus_2	99.86	99.85	99.84	99.85	99.84	99.83	99.83	99.83	99.83	99.85	99.85	99.84	99.84	99.87	99.87	99.91	99.91	99.99	99.96	99.98
33. Kashiwazakivirus_3_6	99.87	99.87	99.85	99.86	99.85	99.84	99.84	99.84	99.84	99.86	99.86	99.84	99.84	99.88	99.88	99.91	99.91	99.96	99.93	99.94
34. Kashiwazakivirus_1	99.87	99.86	99.85	99.85	99.85	99.85	99.85	99.85	99.85	99.85	99.85	99.85	99.85	99.89	99.89	99.92	99.92	99.95	99.93	99.93
35. Kashiwazakivirus 2	99.87	99.86	99.85	99.84	99.84	99.85	99.85	99.85	99.84	99.85	99.85	99.85	99.85	99.89	99.89	99.92	99.92	99.95	99.92	99.93
36. Kashiwazakivirus_4	99.87	99.87	99.85	99.85	99.84	99.85	99.85	99.85	99.85	99.85	99.85	99.85	99.85	99.89	99.90	99.92	99.92	99.95	99.93	99.94
37. Kashiwazakivirus_5	99.87	99.86	99.85	99.85	99.85	99.85	99.85	99.85	99.84	99.85	99.86	99.85	99.85	99.88	99.89	99.92	99.92	99.95	99.92	99.94
38. Brazilian_marseillevirus	99.85	99.84	99.85	99.85	99.84	99.85	99.84	99.85	99.84	99.85	99.85	99.85	99.85	99.88	99.88	99.90	99.90	99.91	99.91	99.90
39. Golden_Marseillevirus	99.79	99.80	99.78	99.79	99.78	99.78	99.78	99.78	99.78	99.80	99.80	99.79	99.79	99.80	99.81	99.83	99.83	99.83	99.83	99.82

	21	22	23	24	25	26	27	28	29	30	31	32	33	34	35	36	37	38	39
1. oosagami_C9	99.87	99.86	99.87	99.87	99.87	99.87	99.87	99.87	99.86	99.86	99.87	99.86	99.87	99.87	99.87	99.87	99.87	99.85	99.79
2. Tokyovirus	99.85	99.84	99.86	99.85	99.85	99.86	99.85	99.85	99.84	99.84	99.85	99.85	99.87	99.86	99.86	99.87	99.86	99.84	99.80
3. Marseillevirus	99.84	99.84	99.86	99.84	99.84	99.85	99.84	99.84	99.84	99.84	99.84	99.84	99.85	99.85	99.85	99.85	99.85	99.85	99.78
4. Melbournevirus	99.85	99.85	99.85	99.85	99.85	99.84	99.85	99.85	99.85	99.84	99.85	99.85	99.86	99.85	99.84	99.85	99.85	99.85	99.79
5. Marseillevirus Shanghai	99.84	99.84	99.85	99.84	99.84	99.84	99.84	99.84	99.84	99.83	99.84	99.84	99.85	99.85	99.84	99.84	99.85	99.84	99.78
6. Cannes_8_virus	99.83	99.83	99.85	99.83	99.83	99.85	99.83	99.83	99.83	99.83	99.84	99.83	99.84	99.85	99.85	99.85	99.85	99.85	99.78
7. Kyotovirus_1	99.83	99.83	99.85	99.83	99.83	99.85	99.83	99.83	99.83	99.83	99.83	99.83	99.84	99.85	99.85	99.85	99.85	99.84	99.78
8. Kyotovirus_2_5	99.83	99.83	99.85	99.83	99.83	99.85	99.83	99.83	99.83	99.83	99.83	99.83	99.84	99.85	99.85	99.85	99.85	99.85	99.78
9. Kyotovirus_3	99.82	99.83	99.85	99.82	99.83	99.84	99.83	99.83	99.83	99.83	99.83	99.83	99.84	99.85	99.84	99.85	99.84	99.84	99.78
10. Kyotovirus_4_6	99.84	99.85	99.85	99.84	99.84	99.84	99.84	99.84	99.85	99.84	99.85	99.85	99.86	99.85	99.85	99.85	99.85	99.85	99.80
11. Kyotovirus_7	99.85	99.85	99.85	99.85	99.85	99.85	99.85	99.85	99.86	99.85	99.85	99.85	99.86	99.85	99.85	99.85	99.86	99.85	99.80
12. sabaishi_A2	99.83	99.84	99.85	99.83	99.84	99.85	99.84	99.84	99.84	99.84	99.84	99.84	99.84	99.85	99.85	99.85	99.85	99.85	99.79
13. yoshiyabu_G4	99.83	99.84	99.85	99.83	99.84	99.85	99.84	99.84	99.84	99.84	99.84	99.84	99.84	99.85	99.85	99.85	99.85	99.85	99.79
14. Tunisvirus_fontaine2	99.87	99.87	99.89	99.86	99.87	99.88	99.87	99.87	99.87	99.88	99.87	99.87	99.88	99.89	99.89	99.89	99.88	99.88	99.80
15. Insectomime virus	99.87	99.87	99.89	99.87	99.87	99.89	99.87	99.87	99.87	99.88	99.88	99.87	99.88	99.89	99.89	99.90	99.89	99.88	99.81
16. Port-miou_virus	99.91	99.91	99.92	99.91	99.91	99.92	99.91	99.91	99.91	99.92	99.91	99.91	99.91	99.92	99.92	99.92	99.92	99.90	99.83
17. Lausannevirus	99.91	99.91	99.92	99.91	99.91	99.92	99.91	99.91	99.91	99.92	99.91	99.91	99.91	99.92	99.92	99.92	99.92	99.90	99.83
18. sabaishi_H11	99.99	99.98	99.95	99.98	99.99	99.95	99.99	99.99	99.98	99.97	99.99	99.99	99.96	99.95	99.95	99.95	99.95	99.91	99.83
19. shinsuna_B6	99.96	99.96	99.93	99.95	99.96	99.92	99.96	99.96	99.96	99.96	99.95	99.96	99.93	99.93	99.92	99.93	99.92	99.91	99.83
20. shinsuna_C7	99.98	99.99	99.93	99.98	99.98	99.93	99.98	99.98	99.99	99.98	99.98	99.98	99.94	99.93	99.93	99.94	99.94	99.90	99.82
21. koaze		99.98	99.94	99.99	99.99	99.94	99.99	99.99	99.98	99.97	99.98	99.99	99.95	99.94	99.94	99.94	99.94	99.90	99.82
22. oosagami_B5	99.98		99.94	99.98	99.98	99.93	99.98	99.98	99.99	99.99	99.97	99.98	99.94	99.94	99.93	99.94	99.94	99.90	99.83
23. oosagami_E6	99.94	99.94		99.94	99.94	99.98	99.94	99.94	99.94	99.94	99.95	99.94	99.97	99.98	99.98	99.97	99.97	99.90	99.84
24. yoshiyabu_A1	99.99	99.98	99.94		99.99	99.94	99.99	99.99	99.98	99.97	99.98	99.99	99.95	99.94	99.94	99.94	99.94	99.90	99.82
25. yoshiyabu C2	99.99	99.98	99.94	99.99		99.94	100	100	99.98	99.97	99.99	99.99	99.95	99.94	99.94	99.95	99.94	99.91	99.82
26. yoshiyabu_C11	99.94	99.93	99.98	99.94	99.94		99.94	99.94	99.93	99.94	99.95	99.95	99.99	99.99	99.99	99.98	99.98	99.90	99.84
27. yoshiyabu_E7	99.99	99.98	99.94	99.99	100	99.94		100	99.98	99.97	99.99	99.99	99.95	99.94	99.94	99.95	99.94	99.91	99.82
28. yoshiyabu_F1	99.99	99.98	99.94	99.99	100	99.94	100		99.98	99.97	99.99	99.99	99.95	99.94	99.94	99.95	99.94	99.91	99.82
29. Noumeavirus	99.98	99.99	99.94	99.98	99.98	99.93	99.98	99.98		99.98	99.98	99.98	99.94	99.94	99.93	99.94	99.94	99.90	99.82
30. Kurlavirus	99.97	99.99	99.94	99.97	99.97	99.94	99.97	99.97	99.98		99.97	99.97	99.94	99.94	99.94	99.93	99.93	99.91	99.83
31. Hokutovirus_1	99.98	99.97	99.95	99.98	99.99	99.95	99.99	99.99	99.98	99.97		99.98	99.96	99.95	99.95	99.95	99.95	99.91	99.82
32. Hokutovirus_2	99.99	99.98	99.94	99.99	99.99	99.95	99.99	99.99	99.98	99.97	99.98		99.95	99.94	99.94	99.95	99.95	99.91	99.83
33. Kashiwazakivirus_3_6	99.95	99.94	99.97	99.95	99.95	99.99	99.95	99.95	99.94	99.94	99.96	99.95		99.99	99.99	99.98	99.98	99.91	99.84
34. Kashiwazakivirus_1	99.94	99.94	99.98	99.94	99.94	99.99	99.94	99.94	99.94	99.94	99.95	99.94	99.99		100	99.99	99.98	99.90	99.84
35. Kashiwazakivirus 2	99.94	99.93	99.98	99.94	99.94	99.99	99.94	99.94	99.93	99.94	99.95	99.94	99.99	100		99.99	99.98	99.90	99.84
36. Kashiwazakivirus_4	99.94	99.94	99.97	99.94	99.95	99.98	99.95	99.95	99.94	99.93	99.95	99.95	99.98	99.99	99.99		99.98	99.90	99.84
37. Kashiwazakivirus_5	99.94	99.94	99.97	99.94	99.94	99.98	99.94	99.94	99.94	99.93	99.95	99.95	99.98	99.98	99.98	99.98		99.90	99.84
38. Brazilian_marseillevirus	99.90	99.90	99.90	99.90	99.91	99.90	99.91	99.91	99.90	99.91	99.91	99.91	99.91	99.90	99.90	99.90	99.90		99.82
39. Golden_Marseillevirus	99.82	99.83	99.84	99.82	99.82	99.84	99.82	99.82	99.82	99.83	99.82	99.83	99.84	99.84	99.84	99.84	99.84	99.82	

Supplementary Figure 1 (Figure S1). Pairwise sequence identity of MCP genes. Pairwise sequence identities are displayed as percentages. The color of each box corresponds to ordered values from high (red) to low (blue).

Methods. The amino acid sequences were aligned using the ClustalW program implemented in the MEGA X software (ver.10.0.3) using default parameters (Kumar et al., 2018). To construct the maximum-likelihood tree, branch support was estimated using 1,000 bootstrap replications, and the Tamura-Nei, 93 (TN93) model was used as a substitution model with a gamma distribution and invariant sites (G+I) (see Fig. 1); moreover, pairwise sequence identity was calculated using MCP gene sequence alignment, as described previously (Aoki et al., 2019).

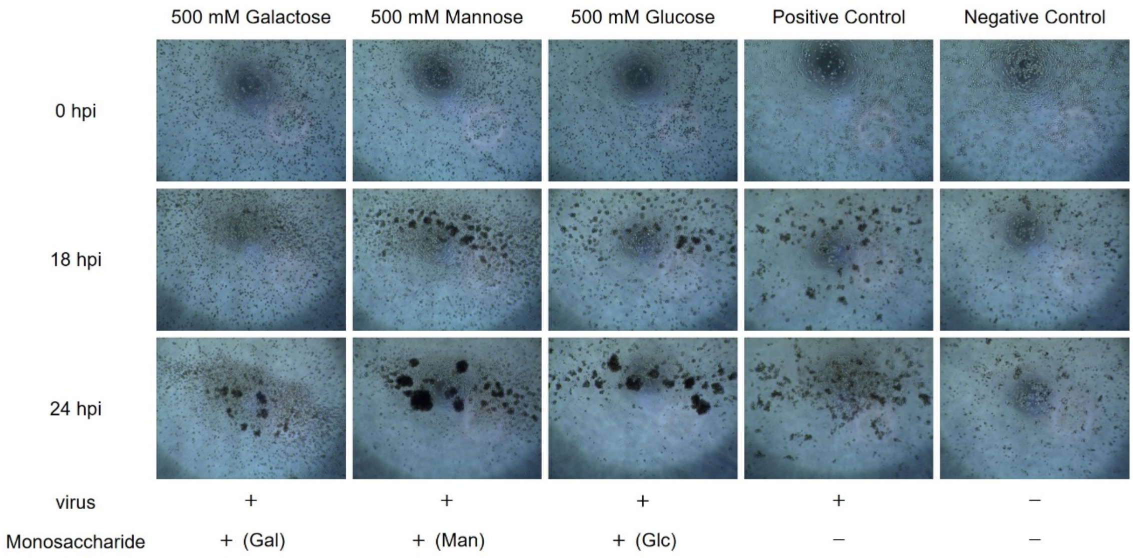

Supplementary Figure 2 (Figure S2). Another experiment on the effects of monosaccharides on "bunch" formation by hokutovirus-infected amoeba cells.

Methods. Hokutovirus-infected amoeba cells were cultured in PY media containing 500 mM galactose, mannose, or glucose. Hokutovirus-infected amoeba cells cultured without any added monosaccharides was used a positive control. Amoeba cells without hokutovirus or any added monosaccharides was used as negative control. Amoeba cells were infected with hokutoviruses (MOI = 1) and observed at 0, 18, and 24 hours post infection (hpi) using an Eclipse TS100 phase-contrast microscope (Nikon, Tokyo, Japan). Gal, galactose; Man, mannose; and Glc, glucose. Objective lens: ×4.

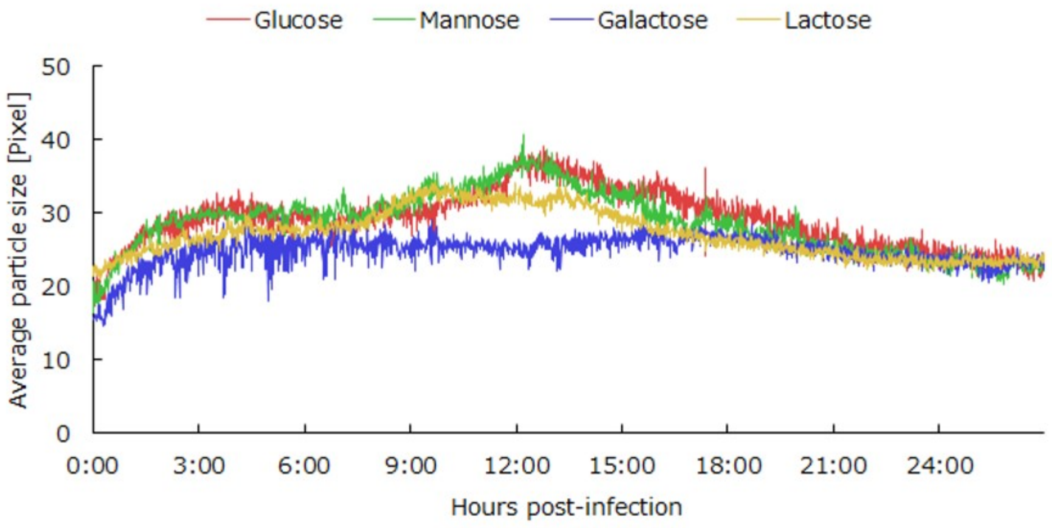

Supplementary Figure 3 (Figure S3). Another kinetic image analysis of hokutovirus-infected amoeba cells in the presence of monosaccharides (glucose, mannose, and galactose) or a disaccharide (lactose) including galactose residue. Bunch formation was inhibited by galactose, but not by lactose, suggesting that the monosaccharide form of galactose was important to inhibit the formation of the bunch.

Methods. Time-lapse images of hokutovirus-infected amoeba cells cultured in media supplemented with 500 mM monosaccharides (glucose, mannose, or galactose) or 500 mM disaccharide (lactose) were captured using BZ-X800/X810 all-in-one fluorescent microscope (Keyence Co.), and kinetic image analyses of cells were performed using the PKA3 algorithm developed in our laboratory, as previously described (Fukaya et al., 2020).

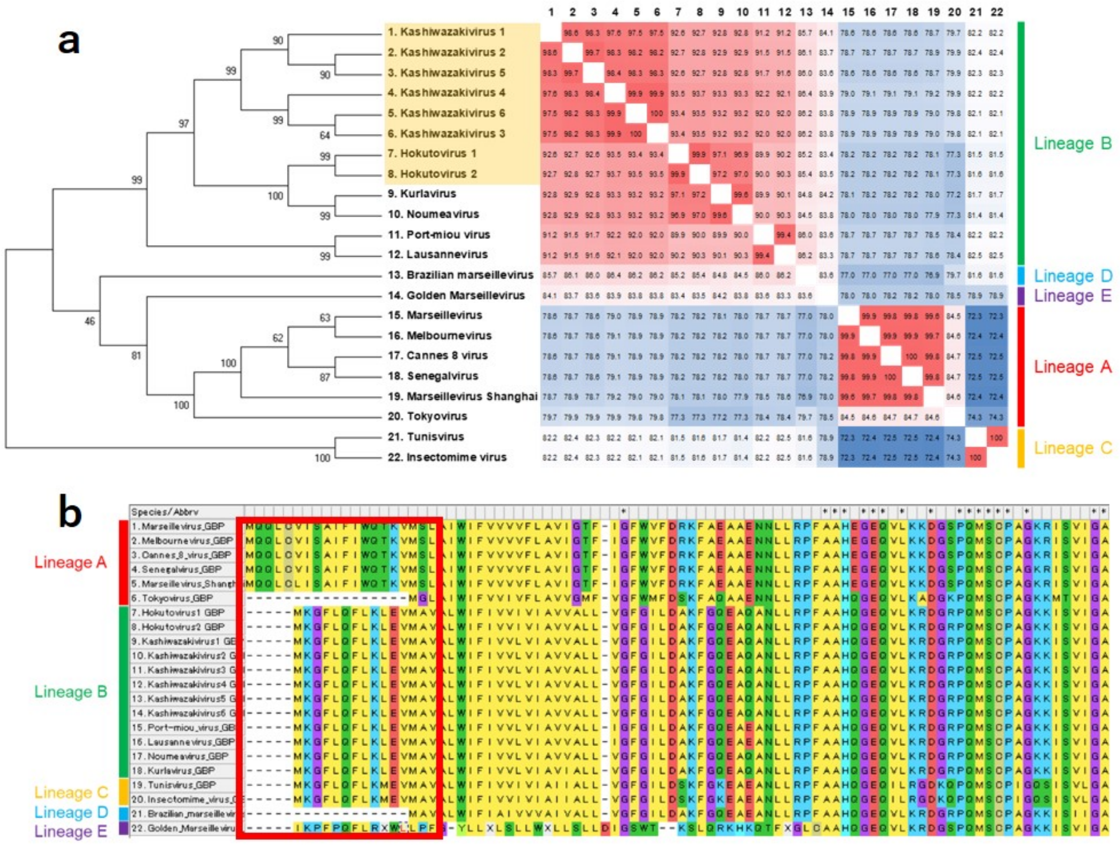

Supplementary Figure 4 (Figure S4). Comparison of *Marseilleviridae* galactose-binding protein (GBP) amino acid sequences. (a) Pairwise sequence identity of GBP genes. Pairwise sequence identities are displayed as percentages. The color of each box corresponds to ordered values from high (red) to low (blue). (b) Alignment of GBP sequences using MEGA X software (Kumar et al., 2018). The red square indicates different sequences between *Marseilleviridae* linage A and lineage B viruses.

Methods. Amino acid sequences were aligned using the ClustalW program implemented in MEGA X software (ver.10.0.3), using default parameters (Kumar et al., 2018). To construct the maximum-likelihood tree, branch support was estimated using 1,000 bootstrap replications, and the Tamura-Nei, 93 (TN93) model was used as a substitution model with a gamma distribution and invariant sites (G+I), moreover, pairwise sequence identity was calculated using GBP gene sequence alignment, as described previously (Aoki et al., 2019).

Supplementary Movie 1 (Movie S1). Part of analyzed time-lapse movie for the kinetic analysis of hokutovirus-infected amoeba cells under conditions of additional **glucose**.
Analyzed particles are outlined in Red and the 12 min tracks of each particle are indicated by blue lines. One second of the movie corresponds to 1 hour. Analytical results of this movie are shown in Fig. 2 of the main text.

Supplementary Movie 2 (Movie S2). Part of analyzed time-lapse movie for the kinetic analysis of hokutovirus-infected amoeba cells under conditions of additional **galactose**.
Analyzed particles are outlined in Red and the 12 min tracks of each particle are indicated by blue lines. One second of the movie corresponds to 1 hour. Analytical results of this movie are shown in Fig. 2b of the main text.
